# Four-Objective Optimization of an Irreversible Stirling Heat Engine with Linear Phenomenological Heat-Transfer Law

**DOI:** 10.3390/e24101491

**Published:** 2022-10-19

**Authors:** Haoran Xu, Lingen Chen, Yanlin Ge, Huijun Feng

**Affiliations:** 1 Institute of Thermal Science and Power Engineering, Wuhan Institute of Technology, Wuhan 430205, China; 2 Hubei Provincial Engineering Technology Research Center of Green Chemical Equipment, Wuhan 430205, China; 3 School of Mechanical & Electrical Engineering, Wuhan Institute of Technology, Wuhan 430205, China

**Keywords:** irreversible Stirling heat engine, finite time thermodynamics, mechanical efficiency theory, linear phenomenological heat-transfer law, mechanical losses, multi-objective optimization

## Abstract

This paper combines the mechanical efficiency theory and finite time thermodynamic theory to perform optimization on an irreversible Stirling heat-engine cycle, in which heat transfer between working fluid and heat reservoir obeys linear phenomenological heat-transfer law. There are mechanical losses, as well as heat leakage, thermal resistance, and regeneration loss. We treated temperature ratio x of working fluid and volume compression ratio λ as optimization variables, and used the NSGA-II algorithm to carry out multi-objective optimization on four optimization objectives, namely, dimensionless shaft power output ${\bar{P}}_{s}$, braking thermal efficiency $\eta_{s}$, dimensionless efficient power ${\bar{E}}_{p}$ and dimensionless power density ${\bar{P}}_{d}$. The optimal solutions of four-, three-, two-, and single-objective optimizations are reached by selecting the minimum deviation indexes D with the three decision-making strategies, namely, TOPSIS, LINMAP, and Shannon Entropy. The optimization results show that the D reached by TOPSIS and LINMAP strategies are both 0.1683 and better than the Shannon Entropy strategy for four-objective optimization, while the Ds reached for single-objective optimizations at maximum ${\bar{P}}_{s}$, $\eta_{s}$, ${\bar{E}}_{p}$, and ${\bar{P}}_{d}$ conditions are 0.1978, 0.8624, 0.3319, and 0.3032, which are all bigger than 0.1683. This indicates that multi-objective optimization results are better when choosing appropriate decision-making strategies.

## 1. Introduction

Finite time thermodynamics (FTT) [1,2,3,4,5,6,7,8,9,10,11,12,13,14,15,16,17,18,19,20,21,22,23,24,25,26,27,28,29,30] has developed and emerged since the mid-1970s. With the continuous development and improvement of the theory, its research objects have expanded from Carnot heat engine to almost all engineering thermal devices and systems, which include the Stirling heat engine (SHE). Blank et al. [31] considered the finiteness of temperature difference between working fluid (WF) and heat reservoir to establish a FTT model of an endoreversible SHE cycle and optimized its power output (P). Chen et al. [32,33] studied SHE with imperfect regeneration, analyzed the influence of regeneration loss, and reached thermal efficiency (η) at maximum P [32], then obtained the η bound of the solar-driven SHE cycle [33]. Wu et al. [34] studied SHE cycle with heat-transfer (HT) loss and regeneration loss, and derived the expressions of cycle P and η. Tlili et al. [35,36] studied the influences of relevant parameters on η of a SHE cycle at maximum P, and found that increasing specific heat capacity of heat sink would cause the SHE cycle to have higher P. Li et al. [37] performed optimization on P of a solar-powered SHE cycle and reached the η at maximum P. Ahmadi et al. [38] studied the influences of heat-exchanger and regenerator parameters on P and η of a SHE cycle. Ahmed et al. [39] studied the influences of HT and flow frictions of a regenerator, a heater, and a cooler on P and η of a beta-type SHE cycle. Ramachandran et al. [40] studied the influences of different types of WF and regenerator materials on cycle P and η of a solar low-temperature differential SHE cycle with regeneration loss. Ahadi et al. [41] studied the influences of coating type and thickness on P and η of a SHE cycle, and pointed out that for different types of coating, η was enhanced with increased coating thickness. De Moura et al. [42] studied the influences of regenerator efficiency, compression ratio, HT coefficient, engine frequency, piston stroke, and area on performance of a space power SHE cycle, and optimized the P and η. Purkait and Biswas [43] studied the effect on P and η of a quantum SHE cycle. Kitaya and Isobe [44] optimized the effect on P and η of a nano-scale β-type SHE cycle.

Power density (PD) was first proposed by Sahin et al. [45], and they used it to perform optimization of a Joule-Brayton cycle. The research showed that compared with the maximum P conditions, the heat engine had smaller dimensions and higher η at maximum PD optimization. Chen et al. [46] studied an endoreversible closed Brayton cycle with thermal resistance, and derived the expression of PD. Ust [47] compared the η of an Atkinson heat-engine cycle with temperature ratio and internal irreversibility loss at maximum P and maximum PD. Gonca [48] studied the influences of internal irreversibility loss and HT loss on a dual-Atkinson cycle, and derived its maximum dimensionless P, η, and dimensionless PD. Karakurt et al. [49] performed optimization on the PD characteristics of a supercritical CO_2_ Brayton cycle.

When studying and optimizing the performance of heat engines, one can neither pursue η and ignore P, nor consider P without considering η. Therefore, in addition to the basic output rate, Yan [50] used a product of cycle P and η (Pη) as an optimization objective to perform optimization on an endoreversible Carnot heat-engine cycle. Yilmaz [51] named it efficient power (EP) and pointed out that a heat engine designed at the maximum EP conditions may have better P than maximum PD optimization. Besides, the maximum EP optimization had a significant η advantage with respect to maximum P optimization. Later, more and more scholars applied it to various heat-engine cycles on the basis of EP optimization objective (OO) [52,53,54,55].

With the increase in OOs, conflicts will occur among various OOs; single-objective optimization will improve one OO but worsen another. In order to take more OOs into account and reach the optimal design scheme, one must perform multi-objective optimization (MOO) research on different thermodynamic cycles. For SHE cycles, Ahmadi et al. [56,57,58] had conducted extensive MOO research on different SHE cycles. Luo et al. [59] took power loss, P, and η as OOs to perform MOO research on SHE cycles, and the results showed that MOO not only improved the P and η, but also significantly reduced the power loss caused by flow resistance. Punnathanam and Kotecha [60] took entropy generation rate, P, and η as OOs to perform MOO research on SHE cycles. Hooshang et al. [61] applied third-order thermodynamic analysis to optimize the performance of SHE cycles, performed MOO research on the cycle with two OOs of P and regenerator differential pressure, and compared the results reached by three decision-making strategies. Dai et al. [62] performed MOO research on a regenerative SHE cycle with three OOs of ecological coefficient of performance, P, and η. Ye et al. [63] took P, η, and exergy efficiency as OOs to perform MOO research on SHE cycles. Shah et al. [64] considered volume ratio, temperature ratio, and surface area ratio of nanoscale SHE cycles, and performed MOO research on the cycles’ ecological coefficient of performance, η and entropy generation rate. Shakouri et al. [65] performed MOO research on solid oxide fuel cell-SHE cycles with three OOs of P, exergy efficiency, and exergy destruction density. Ahmed et al. [66] considered such parameters as heat-source temperature, engine frequency, average effective pressure, piston diameter, and regenerator grid line diameter, and took P, η, and losses as OOs to perform MOO on SHE cycles.

Senft [67,68] proposed mechanical efficiency theory, offering the upper limit of the mechanical efficiency of heat engines, and pointed out that the ideal SHE has maximum mechanical efficiency in the reciprocating heat engine; then, he combined it with FTT to establish a new SHE cycle model which was different from the conventional FTT model. He derived expressions of shaft power output ($P_{s}$) and braking thermal efficiency ($\eta_{s}$), and analyzed the effect of mechanical losses and HT loss with Newtonian heat-transfer law (HTL) (q∝ΔT). On the basis of the model established by Senft [67,68], Xu et al. [69] proposed dimensionless shaft power output (${\bar{P}}_{s}$), $\eta_{s}$, dimensionless EP (${\bar{E}}_{p}$), and dimensionless ecological function, then took them as OOs to perform MOO research on SHE cycles.

Actually, the heat transfer between WF and heat reservoir does not completely obey Newton’s HTL. When options governed by the HTL change, the performance of the heat engine will also change. Therefore, in addition to Newton’s HTL, some scholars have studied the influences of linear phenomenological, radiation, and generalized radiation HTLs on P and η of the endoreversible heat engine [70,71,72]. The authors of references [73,74] studied P and η of an endoreversible Carnot heat engine with generalized convection HTL. Chen et al. [75] studied the maximum P and maximum η of an irreversible Carnot heat engine based on a universal HTL $q\propto {\left(\Delta T^{n}\right)}^{m}$. Li and Chen [76] and Chen and Xia [77] found the optimal configuration of heat engines with $q\propto {\left(\Delta T^{n}\right)}^{m}$ [76] and more universal HTL [77]. Ding et al. [78] optimized the $P_{s}$ and $\eta_{s}$ characteristics of irreversible SHE cycles with linear phenomenological HTL.

On the basis of references [67,68], this study will analyze the effects of mechanical losses, as well as heat leakage, regeneration loss, and thermal resistance on SHE cycles with linear phenomenological HTL ($q\propto \Delta (T^{-1})$). The temperature ratio (x) of the WF and volume compression ratio (λ) of the cycle will be selected as optimization variables, then the NSGA-II algorithm [79,80,81,82] will be applied to perform MOO on four OOs, that is, ${\bar{P}}_{s}$, $\eta_{s}$, ${\bar{E}}_{p}$, and dimensionless PD (${\bar{P}}_{d}$). The Pareto optimal solution of four-, three-, two-, and single-objective optimizations will be reached, and the optimal scheme will be reached by selecting the minimum deviation indexes (D) [83] with TOPSIS [84,85,86], LINMAP [87,88], and Shannon Entropy [89,90] decision-making strategies.

Compared with the previous MOO research of different SHE cycles [56,57,58,59,60,61,62,63,64,65,66,69], the major contribution of this paper is that, firstly, the effects of the linear phenomenological HTL, which is different from Newton’s HTL, on the performance of the SHE are studied, and the expressions of four OOs are derived. It is also found that $P_{s}$, $\eta_{s}$, and $E_{p}$ are obviously different from those in reference [69] referring to Newton’s HTL; secondly, a more realistic cycle model with various heat and mechanical losses is adopted; and, finally, different OOs are introduced. In addition to ${\bar{P}}_{s}$, $\eta_{s}$, and ${\bar{E}}_{p}$, this paper takes ${\bar{P}}_{d}$ as the fourth OO, so the optimization results will be significantly different from the previous research.

## 2. Model of SHE Cycle and OOs

**Figure 1:**
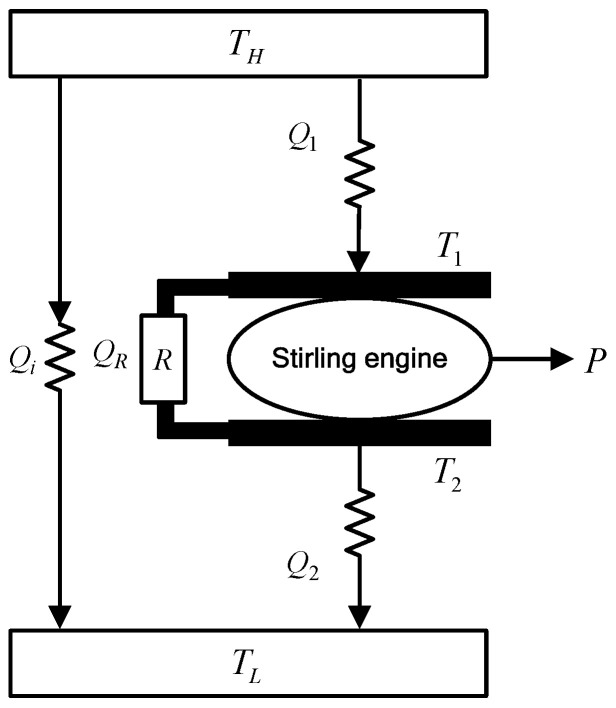
Irreversible SHE cycle model.

An irreversible SHE cycle model [67] is presented in Figure 1. $T_{H}$ and $T_{L}$ are temperatures of heat source and heat sink, WF of the cycle is an ideal gas, R is a regenerator, $Q_{i}$ is heat leakage, $T_{1}$ is temperature of WF in the expansion process, and $T_{2}$ is the temperature of WF in the compression process.

On the basis of thermodynamic properties of ideal gas and linear phenomenological HTL ($q\propto \Delta (T^{-1})$), the heats transferred between the heat reservoir and the WF can be expressed as follows:

(1) $$Q_{1}=\alpha (\frac{1}{T_{1}}-\frac{1}{T_{H}})t_{1}=nR_{u}T_{1}\ln\lambda$$

(2) $$Q_{2}=\beta (\frac{1}{T_{L}}-\frac{1}{T_{2}})t_{2}=nR_{u}T_{2}\ln\lambda$$

where α and β are HT coefficients, $t_{1}$ and $t_{2}$ are time duration of the expansion and compression process, n is mole number of the WF, $\lambda =v_{2}/v_{1}$ is volume compression ratio (equal to the maximum specific volume ratio of the cycle), and $R_{u}$ is the universal gas constant of WF.

The regeneration loss ($\Delta Q_{R}$) of the cycle can be expressed as:

(3) $$\Delta Q_{R}=nC_{v}(1-\eta_{R})(T_{1}-T_{2})$$

where $\eta_{R}$ is the efficiency of the regenerator and $C_{v}$ is the constant volume specific heat capacity of WF.

Sorting out Equations (1) and (2), $t_{1}$ and $t_{2}$ can be expressed as:

(4) $$t_{1}=\frac{nR_{u}T_{1}\ln\lambda }{\alpha (\frac{1}{T_{1}}-\frac{1}{T_{H}})},\;t_{2}=\frac{nR_{u}T_{2}\ln\lambda }{\beta (\frac{1}{T_{L}}-\frac{1}{T_{2}})}$$

The WF temperature (T) varies uniformly with time (t) during the regeneration process, and satisfies the following equation:

(5) $$\frac{dT}{dt}=\pm K_{1}$$

where “+” indicates the heating process and “−” indicates the cooling process; $K_{1}$ ($K_{1}>0$) is only determined by the material of the regenerator.

By integrating Equation (5), the time durations of the regenerative process ($t_{3}$ and $t_{4}$) can be expressed as:

(6) $$t_{3}=\frac{\left(T_{1}-T_{2}\right)}{K_{1}}=t_{4}$$

According to Equations (4) and (6), the cycle period τ can be expressed as:

(7) $$\tau =\frac{nR_{u}T_{1}\ln\lambda }{\alpha (T_{1}{}^{-1}-T_{H}{}^{-1})}+\frac{nR_{u}T_{2}\ln\lambda }{\beta (T_{L}{}^{-1}-T_{2}{}^{-1})}+\frac{2(T_{1}-T_{2})}{K_{1}}$$

The heat leakage can be expressed as:

(8) $$Q_{i}=C_{i}(\frac{1}{T_{L}}-\frac{1}{T_{H}})\tau$$

where $C_{i}$ is the heat-leakage coefficient.

According to Equations (1)–(3) and (8), the heat ($Q_{H}$) supplied by the heat source and heat ($Q_{L}$) released to the heat sink can be expressed as, respectively:

(9) $$Q_{H}=Q_{1}+Q_{i}+\Delta Q_{R}$$

(10) $$Q_{L}=Q_{2}+Q_{i}+\Delta Q_{R}$$

The P and η can be expressed as:

(11) $$P=\frac{Q_{H}-Q_{L}}{\tau }=\frac{\left(Q_{1}-Q_{2}\right)}{\tau }$$

(12) $$\eta =\frac{Q_{H}-Q_{L}}{Q_{H}}=\frac{Q_{1}-Q_{2}}{Q_{1}+Q_{i}+\Delta Q_{R}}$$

According to the expressions of $Q_{H}$, $Q_{L}$, $\Delta Q_{R}$, $Q_{i}$, and τ, the P and η can be further expressed as:

(13) $$P=\frac{n\alpha R_{u}\ln\lambda K_{1}(1-x)(T_{2}-T_{L})(xT_{H}-T_{2})}{2\alpha (1-x)(T_{2}-T_{L})(xT_{H}-T_{2})+nR\ln\lambda K_{1}T_{2}[T_{2}(T_{H}-\delta^{2}xT_{L})+T_{H}T_{L}(\delta^{2}x^{2}-1)]}$$

(14) $$\eta =\frac{n\alpha R_{u}\ln\lambda K_{1}T_{H}T_{L}(1-x)(T_{2}-T_{L})(xT_{H}-T_{2})}{\begin{array}{l} \{2\alpha C_{i}(T_{H}-T_{L})(x-1)(T_{2}-T_{L})(xT_{H}-T_{2})-nR_{u}\ln\lambda K_{1}T_{2}C_{i}(T_{H}-T_{L})[T_{2}(T_{H}-\delta^{2}xT_{L})+\\ T_{H}T_{L}(\delta^{2}x^{2}-1)]+n\alpha K_{1}T_{H}T_{L}(T_{2}-T_{L})(T_{2}-xT_{H})[R_{u}\ln\lambda +C_{v}(x-1)(\eta_{R}-1)]\} \end{array}}$$

where $\delta =\sqrt{\alpha /\beta }$, and $x=T_{2}/T_{1}$ is the WF temperature ratio during the isothermal process.

According to references [30,31,32,33,34], the PD can be expressed as:

(15) $$P_{d}{}^{\prime }=\frac{P}{v_{\max}}=\frac{P}{v_{2}}=\frac{P}{\lambda v_{1}}$$

The optimal temperature for optimal P, η, and $P_{d}{}^{\prime }$ can be expressed as follows:

(16) $$T_{2,opt}=\frac{T_{H}T_{L}[T_{H}(1+\delta x)-T_{L}\delta (1+\delta )]}{T_{H}{}^{2}-\delta T_{L}{}^{2}}$$

Taking the expression of $T_{2,opt}$ into Equations (13)–(15), the optimal performance expressions of P, η, and $P_{d}{}^{\prime }$ can be expressed as:

(17) $$P=\frac{nR_{u}\ln\lambda K_{1}(-T_{H}{}^{2}\delta^{-1}+2T_{H}T_{L}-\delta T_{L}{}^{2})(xT_{H}-T_{L})}{\begin{array}{l} \{2[2T_{H}T_{L}(xT_{H}-T_{L})-(xT_{H}{}^{3}\delta^{-1}-\delta T_{L}{}^{3})-T_{H}T_{L}(\delta xT_{L}-T_{H}\delta^{-1})]-\\ nR_{u}\ln\lambda K_{1}T_{H}T_{L}(T_{H}{}^{2}\delta^{-1}-2T_{H}T_{L}+\delta T_{L}{}^{2})(\delta x^{2}+2x+\delta^{-1})\} \end{array}}$$

(18) $$\eta =\frac{n\alpha R_{u}\ln\lambda K_{1}T_{H}T_{L}(1-x){\left(xT_{H}-T_{L}\right)}^{2}(T_{H}-\delta T_{L})(\delta^{-1}T_{H}-T_{L})}{\begin{array}{l} \{2\alpha C_{i}(T_{H}-T_{L})(x-1)\{x{\left(\delta^{-1}T_{H}{}^{2}-\delta T_{L}{}^{2}\right)}^{2}+{\left[(\delta^{-1}+x)T_{H}-(x\delta +1)T_{L}\right]}^{2}T_{H}T_{L}+[(\delta^{-1}+x)T_{H}-\\ (x\delta +1)T_{L}](\delta^{-1}T_{H}{}^{2}-\delta T_{L}{}^{2})(T_{L}+x\alpha^{2}\delta^{-2}T_{H})\}+nR_{u}\ln\lambda K_{1}T_{H}T_{L}C_{i}(T_{H}-T_{L})[(\delta^{-1}+x)T_{H}-\\ (x\delta +1)T_{L}]\{[(\delta^{-1}+x)T_{H}-(x\delta +1)T_{L}](T_{H}-x\delta^{2}T_{L})+(T_{H}{}^{2}-\delta^{2}T_{L}{}^{2})(\delta^{-1}-\delta x^{2})\}-\\ n\alpha K_{1}T_{H}T_{L}(\delta T_{L}-T_{H})(\delta^{-1}T_{H}-T_{L}){\left(xT_{H}-T_{L}\right)}^{2}[R_{u}\ln\lambda +C_{v}(x-1)(\eta_{R}-1)]\} \end{array}}$$

(19) $$P_{d}{}^{\prime }=\frac{nR_{u}\ln\lambda K_{1}(-T_{H}{}^{2}\delta^{-1}+2T_{H}T_{L}-\delta T_{L}{}^{2})(xT_{H}-T_{L})}{\begin{array}{l} v_{1}\lambda \{2[2T_{H}T_{L}(xT_{H}-T_{L})-(xT_{H}{}^{3}\delta^{-1}-\delta T_{L}{}^{3})-T_{H}T_{L}(\delta xT_{L}-T_{H}\delta^{-1})]-\\ nR_{u}\ln\lambda K_{1}T_{H}T_{L}(T_{H}{}^{2}\delta^{-1}-2T_{H}T_{L}+\delta T_{L}{}^{2})(\delta x^{2}+2x+\delta^{-1})\} \end{array}}$$

**Figure 2:**
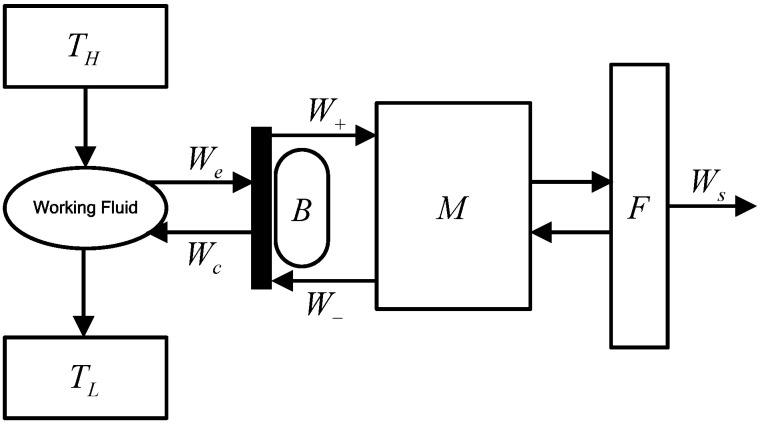
Reciprocating heat-engine model.

A reciprocating heat-engine model is presented in Figure 2 [68], and the arrow indicates the direction of the work transfer. The mechanical device, flywheel, and buffer space are represented by M, F, and B, respectively. The atmosphere often serves as the buffer gas in B. Buffer gas acts on the piston directly and it absorbs and stores energy and returns it to the WF. The arrow indicates the direction of the work transfer. $W_{e}$ and $W_{c}$ are cycle expansion work and compression work. $W_{+}$ is the work conducted by the piston on M, $W_{-}$ is the work conducted by M on the piston. $W_{s}$ is the cycle shaft work, which is also the useful output work produced by the engine in each cycle. The quantity of the output work for the cycle is determined by mechanism effectiveness (e). The ratio of shaft work to indicated work is referred to as mechanical efficiency ($\eta_{ms}$), and Senft pointed out that it cannot exceed as follows for fixed x, λ, and e [68]:

(20) $$\eta_{ms}(e,x,\lambda )=e-S(x,\lambda )(\frac{1}{e}-e)\;\;$$

where:

(21) $$S(x,\lambda )=\;\left\{ \begin{array}{l} \;\;\;\;\;\;0\;\;\;\;\;\;\;\;x\lambda \leq 1\\ \frac{x\ln x-(1+x)[\ln(1+x)-\ln(1+\lambda )]-\ln\lambda }{(1-x)\ln\lambda }\;\;x\lambda >1 \end{array}\right.$$

Combining Equations (17), (18) and (20), the $P_{s}$ and $\eta_{s}$ expressions for the SHE cycle are, respectively:

(22) $$P_{s}=P\eta_{ms}$$

(23) $$\eta_{s}=\eta \eta_{ms}$$

Combining Equations (19)–(23), the EP and PD of the SHE cycle with mechanical losses can be expressed as:

(24) $$E_{p}=P_{s}{}_{}\eta_{s}$$

(25) $$P_{d}=P_{d}{}^{\prime }\eta_{ms}$$

Consequently, the ${\bar{P}}_{s}$, ${\bar{E}}_{p}$, and ${\bar{P}}_{d}$ can be expressed as:

(26) $${\bar{P}}_{s}=P_{s}/{\left(P_{s}\right)}_{\max}$$

(27) $${\bar{E}}_{p}=E_{p}/{\left(E_{p}\right)}_{\max}$$

(28) $${\bar{P}}_{d}=P_{d}/{\left(P_{d}\right)}_{\max}$$

## 3. Multi-Objective Optimizations

Problems with two or more OOs are called MOO problems. MOO can improve at least one OO without deteriorating other objectives, and it does not indicate that each OO reaches the maximum. The Pareto optimal solution is the ultimate result of continuous optimization, and the set composed of these solutions is called the Pareto frontier.

**Figure 3:**
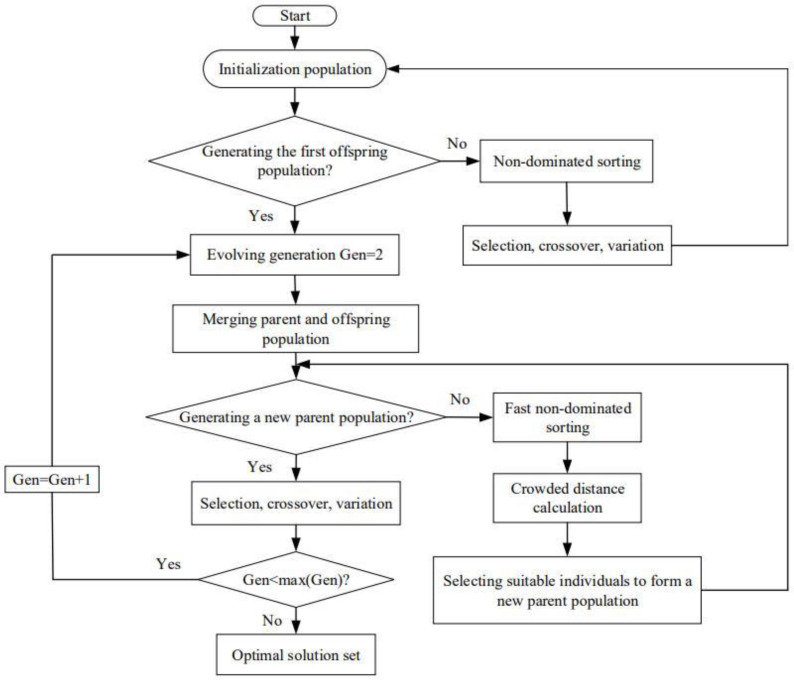
NSGA-II algorithm flow chart.

The NSGA-II algorithm is used to resolve the MOO problem in this paper, and its flow chart is represented as Figure 3. Taking x and λ as optimization variables, and ${\bar{P}}_{s}$, $\eta_{s}$, ${\bar{E}}_{p}$, and ${\bar{P}}_{d}$ are OOs of the cycle, the MOOs are performed on four-, three-, two-, and single-objective by using the NSGA-II algorithm. TOPSIS, LINMAP, and Shannon Entropy decision-making strategies are taken to obtain the optimal scheme by comparing the deviation indexes.

The following parameters are determined by references [67,68]: n=1.0 mol, $C_{v}=20.77\;J/(\mathrm{mol}\cdot K)$, $T_{H}=800\;K$, $T_{L}=300\;K$, and $K_{1}=8.0\times 10^{3}\;K/s$. The value ranges of the two variables are 0.375≤x≤0.775 and 1.15≤λ≤7.15, respectively.

The NSGA-II algorithm’s configuration parameters are listed in Table 1. The results reached by four-, three-, two-, and single-objective optimizations under three strategies are shown in Table 2. According to Table 2, the values of positive ideal points are 1.000, 1.000, 0.3718, and 1.000, respectively, and the values of negative ideal points are 0.3745, 0.1854, 0.4962, and 0.1442, respectively. At the maximum ${\bar{P}}_{s}$, $\eta_{s}$, ${\bar{E}}_{p}$, and ${\bar{P}}_{d}$ conditions, the deviation indexes of four single-objective optimizations are 0.1978, 0.8624, 0.3319, and 0.3032, respectively.

**Figure 4:**
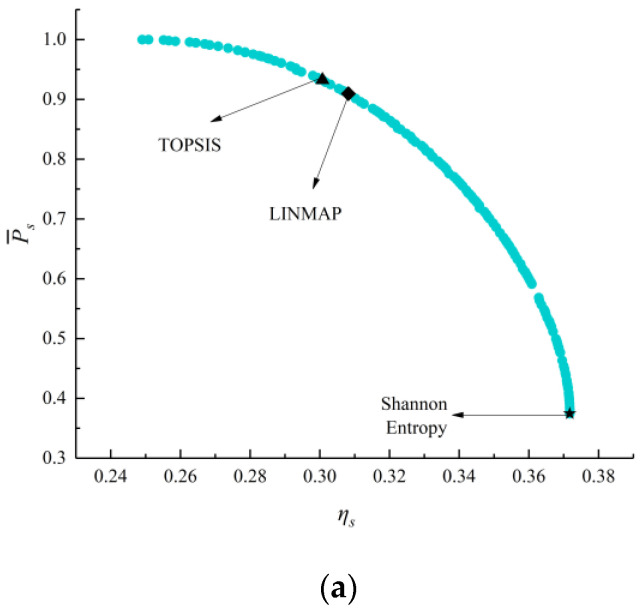

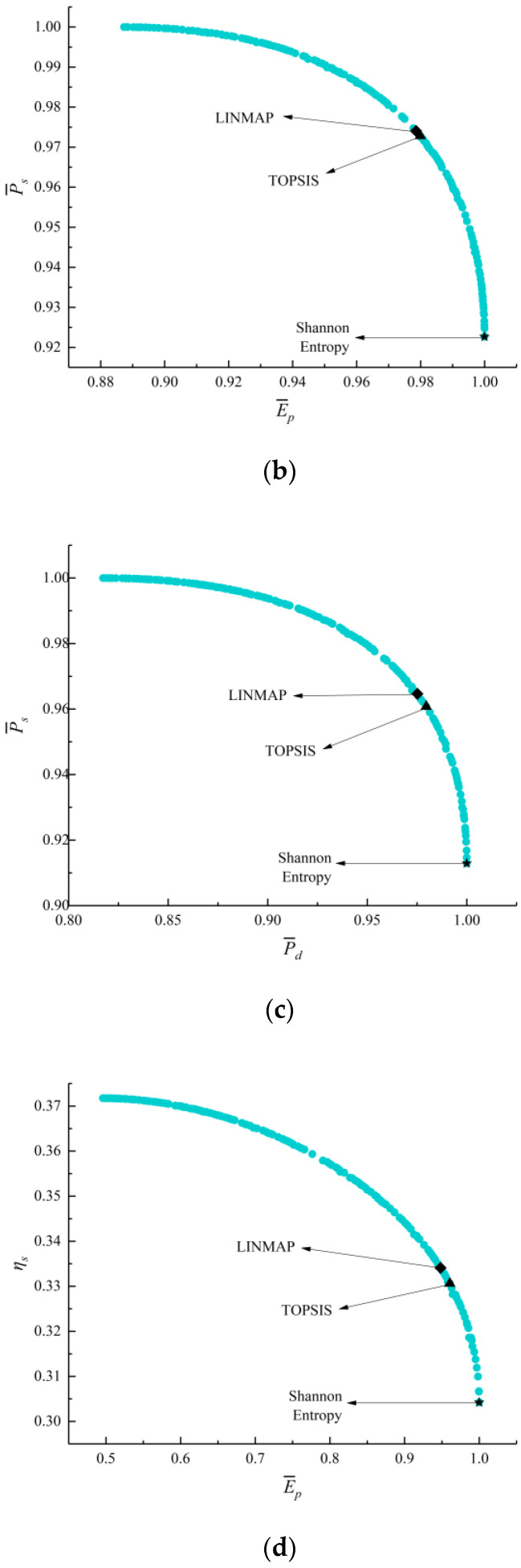

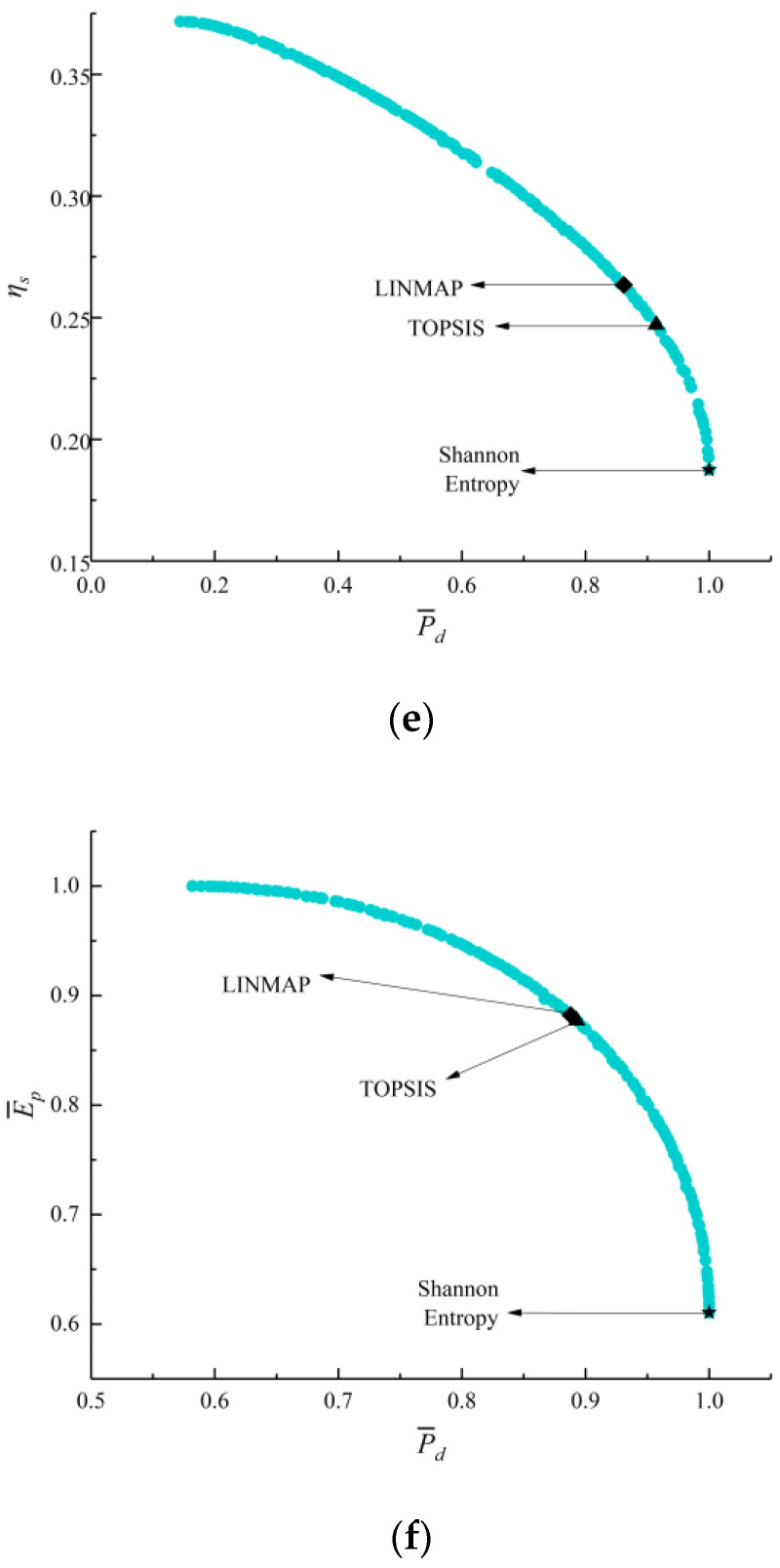
Results for two-objective combinatorial optimizations. (**a**) ${\bar{P}}_{s}-\eta_{s}$ Pareto frontier, (**b**) ${\bar{P}}_{s}-{\bar{E}}_{p}$ Pareto frontier, (**c**) ${\bar{P}}_{s}-{\bar{P}}_{d}$ Pareto frontier, (**d**) $\eta_{s}-{\bar{E}}_{p}$ Pareto frontier, (**e**) $\eta_{s}-{\bar{P}}_{d}$ Pareto frontier, and (**f**) ${\bar{E}}_{p}-{\bar{P}}_{d}$ Pareto frontier.

Figure 4 shows the Pareto frontiers reached by corresponding two-objective optimizations (${\bar{P}}_{s}-\eta_{s}$, ${\bar{P}}_{s}-{\bar{E}}_{p}$, ${\bar{P}}_{s}-{\bar{P}}_{d}$, $\eta_{s}-{\bar{E}}_{p}$, $\eta_{s}-{\bar{P}}_{d}$, and ${\bar{E}}_{p}-{\bar{P}}_{d}$). From these six figures, as ${\bar{P}}_{s}$ grows, $\eta_{s}$, ${\bar{E}}_{p}$, and ${\bar{P}}_{d}$ will all decline. As $\eta_{s}$ grows, ${\bar{E}}_{p}$ and ${\bar{P}}_{d}$ will decline. As ${\bar{E}}_{p}$ grows, ${\bar{P}}_{d}$ will decline. According to Table 2, when MOO is performed on ${\bar{P}}_{s}-\eta_{s}$, the deviation index (0.3250) calculated by the TOPSIS strategy is smaller. When MOO is performed on ${\bar{P}}_{s}-{\bar{E}}_{p}$, ${\bar{P}}_{s}-{\bar{P}}_{d}$, $\eta_{s}-{\bar{P}}_{d}$, and ${\bar{E}}_{p}-{\bar{P}}_{d}$, the deviation indexes (0.2580, 0.2286, 0.1782, and 0.1636) calculated by the LINMAP strategy are smaller. When MOO is performed on $\eta_{s}-{\bar{E}}_{p}$, the deviation index (0.3306) calculated by the Shannon Entropy strategy is smaller.

**Figure 5:**
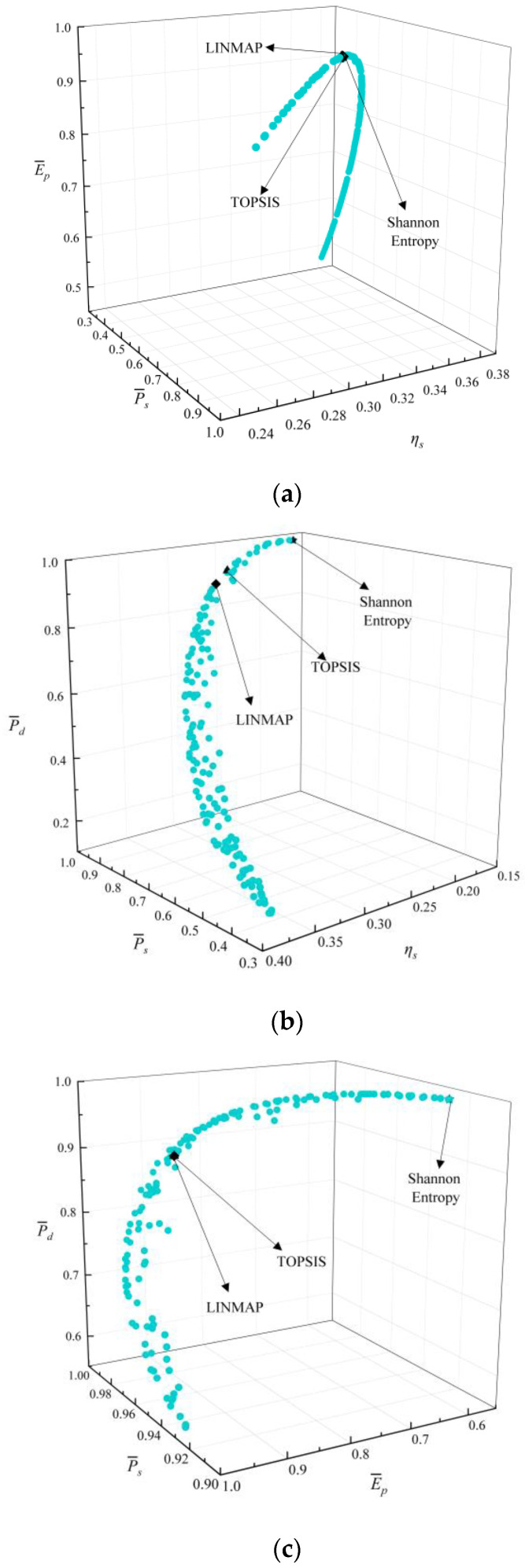

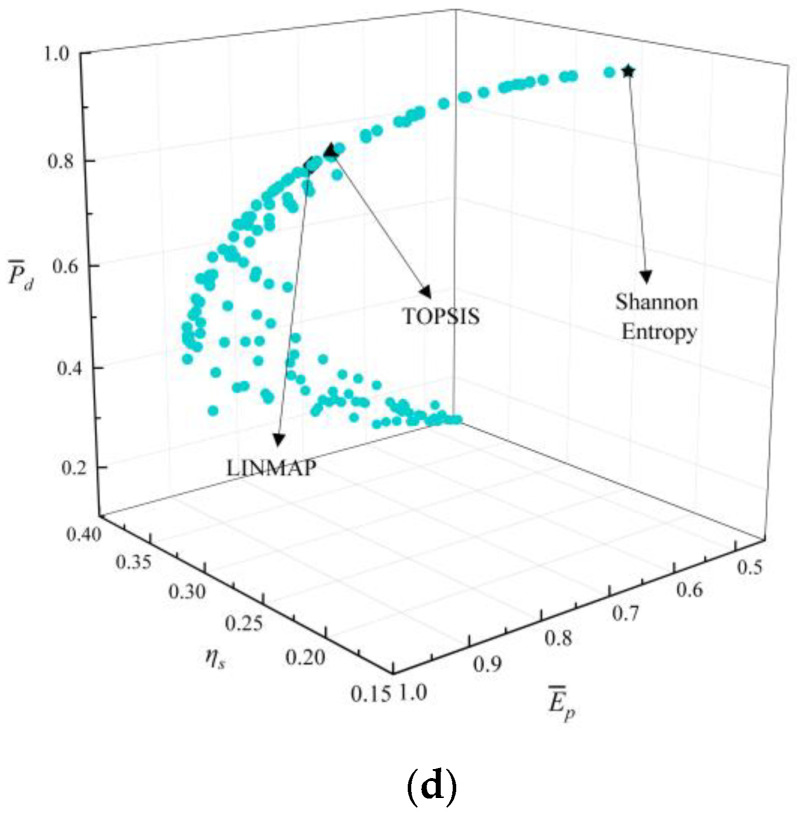
Results for three-objective combinatorial optimizations. (**a**) ${\bar{P}}_{s}-\eta_{s}-{\bar{E}}_{p}$ Pareto frontier, (**b**) ${\bar{P}}_{s}-\eta_{s}-{\bar{P}}_{d}$ Pareto frontier, (**c**) ${\bar{P}}_{s}-{\bar{E}}_{p}-{\bar{P}}_{d}$ Pareto frontier, and (**d**) $\eta_{s}-{\bar{E}}_{p}-{\bar{P}}_{d}$ Pareto frontier.

Figure 5 shows the Pareto frontiers reached by corresponding three-objective optimizations (${\bar{P}}_{s}-\eta_{s}-{\bar{E}}_{p}$, ${\bar{P}}_{s}-\eta_{s}-{\bar{P}}_{d}$, ${\bar{P}}_{s}-{\bar{E}}_{p}-{\bar{P}}_{d}$, and $\eta_{s}-{\bar{E}}_{p}-{\bar{P}}_{d}$). As ${\bar{P}}_{s}$ grows, $\eta_{s}$ will decline, ${\bar{E}}_{p}$ and ${\bar{P}}_{d}$ will all first grow and then decline. As $\eta_{s}$ grows, ${\bar{P}}_{d}$ will decline, ${\bar{E}}_{p}$ will first grow and then decline. According to Table 2, when MOO is performed on ${\bar{P}}_{s}-\eta_{s}-{\bar{E}}_{p}$, the deviation indexes (0.3306) calculated by the TOPSIS and Shannon Entropy strategies are the same and smaller than that (0.3455) reached by the LINMAP strategy. When MOO is performed on ${\bar{P}}_{s}-\eta_{s}-{\bar{P}}_{d}$, the deviation index (0.1648) reached by the LINMAP strategy is smaller. When MOO is performed on $\eta_{s}-{\bar{E}}_{p}-{\bar{P}}_{d}$, the deviation index (0.1663) reached by the TOPSIS strategy is smaller. When MOO is performed on ${\bar{P}}_{s}-{\bar{E}}_{p}-{\bar{P}}_{d}$, the deviation indexes (0.1641) reached by the LINMAP and TOPSIS strategies are the same and smaller.

**Figure 6:**
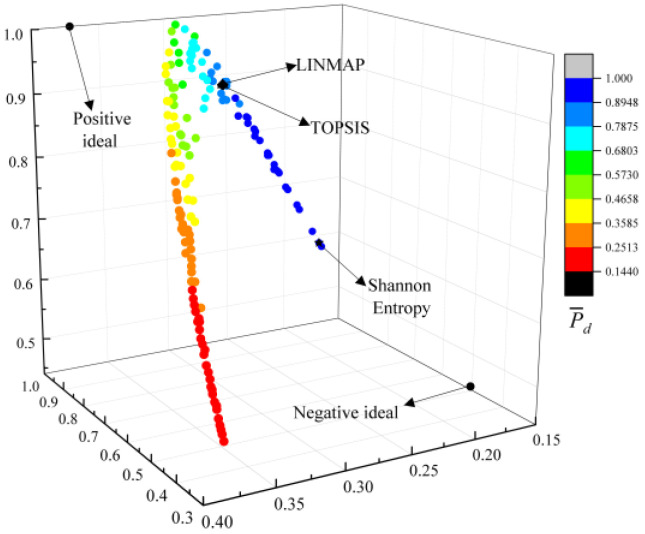
${\bar{P}}_{s}-\eta_{s}-{\bar{E}}_{p}-{\bar{P}}_{d}$ Pareto frontier.

Figure 6 shows the Pareto frontier reached by four-objective optimization (${\bar{P}}_{s}-\eta_{s}-{\bar{E}}_{p}-{\bar{P}}_{d}$). From Figure 6, the three axes stand in for the values of the ${\bar{P}}_{s}$, $\eta_{s}$, and ${\bar{E}}_{p}$, respectively, and the change of the value of ${\bar{P}}_{d}$ is represented by the change in color on the Pareto frontier. The positive and negative ideal points are the points where the four OOs reach the best or worst values at the same time. It can be found that there are no optimal or worst x and λ to make the four OOs all reach the maximum or minimum at the same time. As ${\bar{P}}_{s}$ grows, $\eta_{s}$ will decline, and ${\bar{E}}_{p}$ and ${\bar{P}}_{d}$ will first grow and then decline.

Table 2 shows that when performing MOO on ${\bar{P}}_{s}-\eta_{s}-{\bar{E}}_{p}-{\bar{P}}_{d}$, the deviation indexes reached by TOPSIS and LINMAP strategies are smaller and their results are superior to those of the Shannon Entropy strategy.

**Figure 7:**
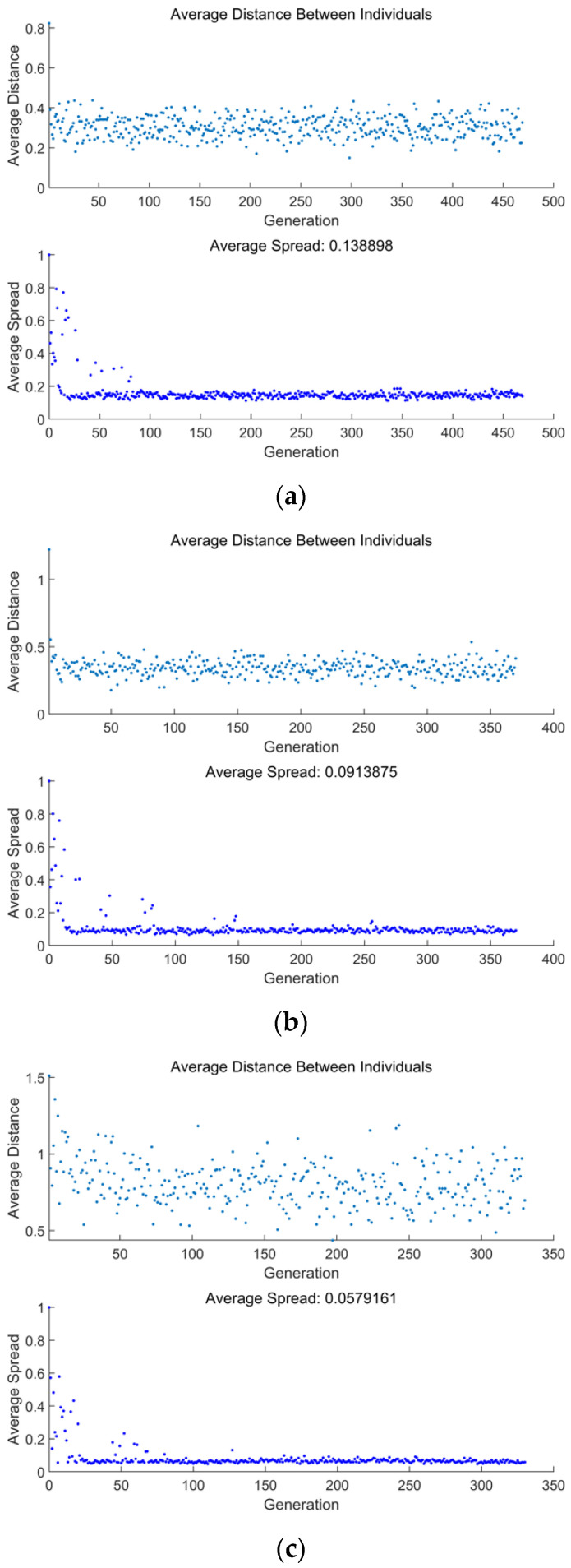
Average distance and spread versus the number of generations. (**a**) ${\bar{E}}_{p}-{\bar{P}}_{d}$, (**b**) ${\bar{P}}_{s}-{\bar{E}}_{p}-{\bar{P}}_{d}$, and (**c**) ${\bar{P}}_{s}-\eta_{s}-{\bar{E}}_{p}-{\bar{P}}_{d}$.

Figure 7 shows the average distance and spread versus the number of generations for three different MOOs (${\bar{E}}_{p}-{\bar{P}}_{d}$, ${\bar{P}}_{s}-{\bar{E}}_{p}-{\bar{P}}_{d}$, and ${\bar{P}}_{s}-\eta_{s}-{\bar{E}}_{p}-{\bar{P}}_{d}$). From Figure 7a–c, when the genetic algorithm approaches convergence, which happens at 470th, 371st, and 331st generations for ${\bar{E}}_{p}-{\bar{P}}_{d}$, ${\bar{P}}_{s}-{\bar{E}}_{p}-{\bar{P}}_{d}$, and ${\bar{P}}_{s}-\eta_{s}-{\bar{E}}_{p}-{\bar{P}}_{d}$ optimizations, respectively, the genetic algorithm ends immediately.

## 4. Conclusions

On the basis of the model established in references [67,68] and the NSGA-II algorithm, this study performs thermodynamic analysis and MOO on an irreversible SHE with linear phenomenological HTL. We treated x and λ as optimization variables, and utilized four performance indicators, namely, ${\bar{P}}_{s}$, $\eta_{s}$, ${\bar{E}}_{p}$, and ${\bar{P}}_{d}$, which were treated as OOs. We utilized TOPSIS, LINMAP, and Shannon Entropy strategies to reach deviation indexes of MOO on different combinations of OOs. The results showed that:

1. From the expressions derived of the four OOs under linear phenomenological HTL it was found that $P_{s}$, $\eta_{s}$, and $E_{p}$ were obviously different from those in reference [69], which indicates that the change of HTL also fundamentally changes the performance indicators of the heat engine;
2. The deviation indexes calculated by TOPSIS and LINMAP decision-making strategies are both 0.1683 when MOO is performed on ${\bar{P}}_{s}-\eta_{s}-{\bar{E}}_{p}-{\bar{P}}_{d}$, which are smaller and the optimization results are better than the results using the Shannon Entropy strategy. Compared with the deviation indexes (0.1978, 0.8624, 0.3319, and 0.3032) calculated by single-objective optimization at maximum ${\bar{P}}_{s}$, $\eta_{s}$, ${\bar{E}}_{p}$, and ${\bar{P}}_{d}$ conditions, the deviation indexes of MOO are smaller and their results are better;
3. When the genetic algorithm approaches convergence, which happens at the 331st generation for ${\bar{P}}_{s}-\eta_{s}-{\bar{E}}_{p}-{\bar{P}}_{d}$ optimization, the genetic algorithm ends immediately. The average distance and spread gradually decrease from the beginning to the 25th generation, after which they remain stable until the end of the calculation. The average distance is mainly between 0.5~1.5, and the average spread keeps to nearly zero after the 25th generation, which suggests that the optimization process is nearly stable;
4. When performing triple-objective optimizations, the MOO results of ${\bar{P}}_{s}-{\bar{E}}_{p}-{\bar{P}}_{d}$ are better than the other combinations. The average distance mainly ranges from 0 to 0.5, and the average spread keeps to nearly zero after the 15th generation. When performing double-objective optimizations, the MOO results of ${\bar{E}}_{p}-{\bar{P}}_{d}$ are better than the other combinations. The average distance mainly ranges from 0.2 to 0.4, and the average spread keeps to nearly zero after the 20th generation;
5. Compared with single-objective optimization, MOO can better take different OOs into account by choosing appropriate decision-making strategies. For the results of different objective combinations, appropriate schemes can be selected according to the actual design and operation to meet the requirements under different working conditions;
6. FTT and MOO are effective tools to guide performance improvement and optimization for SHE cycles. The consideration of different HTLs is also necessary.

## Figures and Tables

**Table 1:** NSGA-II algorithm parameters.

Parameters	Values
Generations	700
Population size	300
Pareto fraction	0.5
Crossover fraction	0.8

**Table 2:** Results of four-, three-, two-, and single-objective optimizations.

Optimization Methods	Decision- Making Strategies	Optimization Variables		Optimization Objectives				Deviation Index
		x	λ	${\bar{P}}_{s}$	$\eta_{s}$	${\bar{E}}_{p}$	${\bar{P}}_{d}$	D
Four-objective optimization $({\bar{P}}_{s}$$,\;\eta_{s}$$,{\bar{E}}_{p}$$,\;\mathrm{and}\;{\bar{P}}_{d}$)	LINMAP	0.5815	1.5301	0.9608	0.2601	0.8905	0.8721	0.1683
	TOPSIS	0.5815	1.5301	0.9608	0.2601	0.8905	0.8721	0.1683
	Shannon Entropy	0.6610	1.2684	0.9128	0.1877	0.6103	1.0000	0.3018
Three-objective optimization $({\bar{P}}_{s}$$,\;\eta_{s}$$,\;\mathrm{and}\;{\bar{E}}_{p}$)	LINMAP	0.5462	2.2788	0.9178	0.3056	0.9995	0.5597	0.3455
	TOPSIS	0.5475	2.2032	0.9226	0.3042	1.0000	0.5819	0.3306
	Shannon Entropy	0.5475	2.2032	0.9226	0.3042	1.0000	0.5819	0.3306
Three-objective optimization $({\bar{P}}_{s}$$,\;\eta_{s}$$,\;\mathrm{and}\;{\bar{P}}_{d}$)	LINMAP	0.5885	1.5360	0.9670	0.2576	0.8903	0.8775	0.1648
	TOPSIS	0.5968	1.4652	0.9658	0.2462	0.8475	0.9161	0.1735
	Shannon Entropy	0.6611	1.2679	0.9124	0.1875	0.6095	1.0000	0.3022
Three-objective optimization $({\bar{P}}_{s}$$,\;{\bar{E}}_{p}$$,\;\mathrm{and}\;{\bar{P}}_{d}$)	LINMAP	0.6030	1.5375	0.9835	0.2512	0.8802	0.8889	0.1641
	TOPSIS	0.6030	1.5375	0.9835	0.2512	0.8802	0.8889	0.1641
	Shannon Entropy	0.6610	1.2686	0.9129	0.1877	0.6106	1.0000	0.3016
Three-objective optimization $(\eta_{s}$$,\;{\bar{E}}_{p}$$,\;\mathrm{and}\;{\bar{P}}_{d}$)	LINMAP	0.5718	1.2686	0.9502	0.2663	0.9017	0.8509	0.1756
	TOPSIS	0.5852	1.5303	0.9653	0.2584	0.8890	0.8766	0.1663
	Shannon Entropy	0.6610	1.2686	0.9129	0.1877	0.6106	1.0000	0.3016
Two-objective optimization $({\bar{P}}_{s}$$\;\mathrm{and}\;\eta_{s}$)	LINMAP	0.5407	2.1898	0.9095	0.3082	0.9988	0.5771	0.3367
	TOPSIS	0.5531	2.2047	0.9325	0.3008	0.9992	0.5877	0.3250
	Shannon Entropy	0.4208	3.6089	0.3745	0.3718	0.4962	0.1442	0.8630
Two-objective optimization $({\bar{P}}_{s}$$\;\mathrm{and}\;{\bar{E}}_{p}$)	LINMAP	0.5793	1.9746	0.9740	0.2820	0.9786	0.6855	0.2580
	TOPSIS	0.5783	1.9812	0.9728	0.2827	0.9799	0.6824	0.2600
	Shannon Entropy	0.5476	2.2031	0.9227	0.3042	1.0000	0.5820	0.3305
Two-objective optimization $({\bar{P}}_{s}$$\;\mathrm{and}\;{\bar{P}}_{d}$)	LINMAP	0.6459	1.3747	0.9647	0.2141	0.7360	0.9752	0.2286
	TOPSIS	0.6468	1.3629	0.9608	0.2120	0.7257	0.9796	0.2345
	Shannon Entropy	0.6610	1.2686	0.9129	0.1877	0.6107	1.0000	0.3016
Two-objective optimization $(\eta_{s}$$\;\mathrm{and}\;{\bar{E}}_{p}$)	LINMAP	0.5026	2.6195	0.7965	0.3341	0.9482	0.4226	0.4700
	TOPSIS	0.5079	2.5508	0.8154	0.3306	0.9605	0.4442	0.4491
	Shannon Entropy	0.5475	2.2033	0.9226	0.3042	1.0000	0.5819	0.3306
Two-objective optimization $(\eta_{s}$$\;\mathrm{and}\;{\bar{P}}_{d}$)	LINMAP	0.5709	1.5216	0.9439	0.2835	0.8861	0.8620	0.1782
	TOPSIS	0.5898	1.4515	0.9551	0.2471	0.8410	0.9144	0.1792
	Shannon Entropy	0.6614	1.2682	0.9126	0.1875	0.6098	1.0000	0.3021
Two-objective optimization $({\bar{E}}_{p}$$\;\mathrm{and}\;{\bar{P}}_{d}$)	LINMAP	0.5989	1.5332	0.9797	0.2527	0.8821	0.8879	0.1636
	TOPSIS	0.5984	1.5217	0.9777	0.2518	0.8772	0.8928	0.1642
	Shannon Entropy	0.6610	1.2686	0.9129	0.1877	0.6106	1.0000	0.3016
$\mathrm{Maximum}\;{\bar{P}}_{s}$	-	0.6229	1.7047	1.0000	0.2501	0.8909	0.8152	0.1978
$\mathrm{Maximum}\;\eta_{s}$	-	0.4213	3.6042	0.3777	0.3718	0.5003	0.1456	0.8624
$\mathrm{Maximum}\;{\bar{E}}_{p}$	-	0.5469	2.2063	0.9213	0.3046	1.0000	0.5803	0.3319
$\mathrm{Maximum}\;{\bar{P}}_{d}$	-	0.6626	1.2676	0.9121	0.1870	0.6078	1.0000	0.3032
Positive ideal point		-	-	1.0000	0.3718	1.0000	1.0000	-
Negative ideal point		-	-	0.3745	0.1854	0.4962	0.1442	-

## Data Availability

Not applicable.

## Nomenclature

B	Buffer space
$C_{i}$	Heat-leakage coefficient, W/K
$C_{v}$	Molar constant volume specific heat capacity, W/K
e	Mechanism effectiveness
F	Flywheel
M	Mechanical device
n	Mole number, mol
R	Regenerator
$R_{u}$	Universal gas constant, J/(mol⋅K)
T	Temperature, K
t	Time duration of the process, s
V	Volume, $m^{3}$
$W_{c}$	Compression work, J
$W_{e}$	Expansion work, J
$W_{+}$	Positive piston work, J
$W_{-}$	Negative piston work, J
Greek symbol	
α, β	Heat-transfer coefficient, W/K
λ	Volume–compression ratio
$\eta_{R}$	Efficiency of the regenerator
τ	Cycle period, s
σ	Entropy-generation rate, W/K
Subscripts	
opt	Optimal
Superscripts	
−	Dimensionless
EP	Efficient power
FTT	Finite time thermodynamics
HT	Heat transfer
HTL	Heat-transfer law
MOO	Multi-objective optimization
OO	Optimization objective
PD	Power density
SHE	Stirling heat engine
WF	Working fluid
